# Integration of Stable Ionic Liquid-Based Nanofluids into Polymer Membranes. Part I: Membrane Synthesis and Characterization

**DOI:** 10.3390/nano11030607

**Published:** 2021-02-28

**Authors:** Carolina Hermida-Merino, Fernando Pardo, Gabriel Zarca, João M. M. Araújo, Ane Urtiaga, Manuel M. Piñeiro, Ana B. Pereiro

**Affiliations:** 1 Centro de Investigaciones Biomédicas (CINBIO), Department of Applied Physics, University of Vigo, E36310 Vigo, Spain; cahermida@uvigo.es (C.H.-M.); mmpineiro@uvigo.es (M.M.P.); 2 Department of Chemical and Biomolecular Engineering, Universidad de Cantabria, 39005 Santander, Spain; pardof@unican.es (F.P.); zarcag@unican.es (G.Z.); urtiaga@unican.es (A.U.); 3 LAQV, REQUIMTE, Departamento de Química, Faculdade de Ciências e Tecnologia, Universidade Nova de Lisboa, 2829-516 Caparica, Portugal; jmmda@fct.unl.pt

**Keywords:** materials for gas separation, ionanofluids, functionalized pebax^®^1657 membrane, thermal and morphological properties

## Abstract

In this work, polymeric membranes functionalized with ionic liquids (ILs) and exfoliated graphene nanoplatelets (xGnP) were developed and characterized. These membranes based on graphene ionanofluids (IoNFs) are promising materials for gas separation. The stability of the selected IoNFs in the polymer membranes was determined by thermogravimetric analysis (TGA). The morphology of membranes was characterized using scanning electron microscope (SEM) and interferometric optical profilometry (WLOP). SEM results evidence that upon the small addition of xGnP into the IL-dominated environment, the interaction between IL and xGnP facilitates the migration of xGnP to the surface, while suppressing the interaction between IL and Pebax^®^1657. Fourier transform infrared spectroscopy (FTIR) was also used to determine the polymer–IoNF interactions and the distribution of the IL in the polymer matrix. Finally, the thermodynamic properties and phase transitions (polymer–IoNF) of these functionalized membranes were studied using differential scanning calorimetry (DSC). This analysis showed a gradual decrease in the melting point of the polyamide (PA6) blocks with a decrease in the corresponding melting enthalpy and a complete disappearance of the crystallinity of the polyether (PEO) phase with increasing IL content. This evidences the high compatibility and good mixing of the polymer and the IoNF.

## 1. Introduction

Reducing emissions of fluorinated gases (F-gases) is at present one of the most urgent environmental issues. Despite being energy efficient, ozone harmless, user safe, with low levels of flammability and toxicity, fluorinated gases are among the most powerful greenhouse gases, with a global warming effect up to 23,000 times greater than that of dioxide of carbon and lifetimes up to 50,000 year in the atmosphere. Then, the F-gases atmospheric emissions must imperatively be reduced, in accordance with the Kyoto protocol, further developed by the Kigali agreement (international agreement specific for these compounds signed in 2016) and the most recent EU regulation [1]. The development of more benign and efficient materials designed according to the principles of green chemistry [2,3] to recover fluorinated gases used in refrigeration and air conditioning [4] and reducing their emissions are the objectives of this study.

Among separation techniques, membrane-based separation processes have received attention for gas capture due to their high energy efficiency, simplicity, and reduced cost. Furthermore, among the various upgrading technologies, membrane separation has attracted much attention for its relatively low energy consumption and small footprint. The design and development of new materials with selective transport pathways and high gas permeability is a practical approach to improve the gas separation performance of these membranes. However, it is still challenging to obtain an optimal interfacial morphology and good dispersion through the polymer matrix [5].

Two-dimensional graphene nanosheets, with tunable surface functionality [6,7], facile synthesis and high mechanical and thermal properties, have been used as a promising filler to develop novel and specific membranes for gas separations [5]. The high aspect ratio of graphene nanosheets provides longer and more tortuous paths for larger gas molecules to pass through the polymer membranes. This property might decrease the permeability of larger gas molecules while efficiently enhancing the overall gas selectivity. Furthermore, the presence of polar groups close to the surface of exfoliated graphene nanoplatelets (xGnP) (e.g., IL) enhances the compatibility of the filler with the polymer matrix [8]. Ionic liquids (ILs) are the subject of increasing research efforts, owing to their unique physico–chemical properties such as a wide liquid range, stability at high temperatures, non-flammability and negligible vapor pressure [9]. These features have contributed to their recognition as ambient friendly media [10], alternatives to the conventional volatile, flammable and toxic organic solvents used in chemical processes [11].

Mass transport is in general much faster in ILs than in polymer materials, thus allowing higher fluxes through the membrane and more effective (‘‘faster’’) separation [12]. ILs can be considered as ‘‘designer solvents’’ for specific applications because their properties can be finely tuned by selecting the anion, the cation and their side functional groups. In virtue of their peculiar characteristics, ILs have been successfully applied also in the preparation of stable membranes for gas separation [13,14,15]. ILs have also been incorporated into porous materials as cavity occupants or binding agents to improve the physicochemical properties and gas affinity [16].

On the other hand, an important aspect of this investigation is the incorporation of xGnP-IL with good dispersibility in the polymeric matrix that is easily applied to the development of thin film membranes. In this work, the functionalized polymeric membranes were prepared by incorporating ionic liquid with graphene (xGnP–IL) in poly (ether–block–amide) a family of elastomeric multiblock copolymers commercially available with the trademark Pebax^®^1657. It has a molecular structure composed of polyether (PEO) amorphous rubbery segments and hard polyamide (PA6) semi-crystalline segments [17]. The properties of these copolymers are related to the relative content of PEO and PA6 and to their chemical characteristics. Pebax^®^1657 block copolymers contain a phase-separated microstructure in which the hard PA6 segments provide mechanical stability and contribute to crystallinity, while only the soft PEO blocks act as a permeable phase owing to their high chain mobility and thus control the gas transport. Among the different products commercially available, Pebax^®^1657 is very hydrophilic, containing 40 wt. % PA6 and 60 wt. % PEO, and it has been widely studied for membrane gas separation applications [18].

The literature review has shown that the introduction of these additives improves the general gas transport properties of the polymeric matrix [5]. However, poor filler-polymer compatibilities and filler aggregation can have a detrimental impact on the mechanical and separation performance of the final membranes [19,20]. In this study, Pebax^®^1657 membranes were coupled with a fluorinated IL and graphene to achieve gas-selective membranes with higher permeability. This work shows for the first time the high compatibility of graphene ionanofluids (IoNFs), based on highly surfactant IL, [21] with the copolymer Pebax^®^1657. The surfactant IL chosen in this work allows to improve the dispersion of the graphene within the polymer matrix, increasing at the same time the mass transport through these new membranes. Additionally, both IL and graphene can increase the selectivity and separating power of these novel materials. Then, the objective of this work is to develop these novel and stable membranes using a simple solution molding technique. Two composite ionic liquid and polymer membranes (Pebax^®^1657 (80 wt% and 60 wt%) + (20 wt% and 40 wt% IL) and six mixed matrix membranes (Pebax^®^1657 (80 wt% and 60 wt%) + (20 wt% and 40 wt% of IoNF previously prepared at three different concentrations of graphene nanoparticles (1 wt%, 10 wt% and 20 wt%)) were prepared and characterized by thermogravimetric analysis (TGA), differential scanning calorimetry (DSC), scanning electron microscope (SEM), interferometric optical profilometry (WLOP) and Fourier transform infrared spectroscopy (FTIR). These different characterization techniques have been applied to determine the morphology, thermal and structural properties in view of the potential use of these membranes.

## 2. Materials and Methods

### 2.1. Materials for IoNF Preparation

Firstly, six different IoNFs were prepared using a highly surfactant fluorinated ionic liquid (FIL) as base fluid (base fluids for use in gas separation) and they were functionalized with pristine graphene, xGnP. Nano-powder was weighed with a Mettler AE-240 balance, whose accuracy is estimated to be 5 × 10^−5^ g, and then dispersed into a calculated volume of the base fluid, obtaining stable and homogeneous xGnP/FIL (IoNFs), with percent weight concentrations of 0.2, 0.4, 2, 4, 8 and 12 wt%.

**Figure 1:**
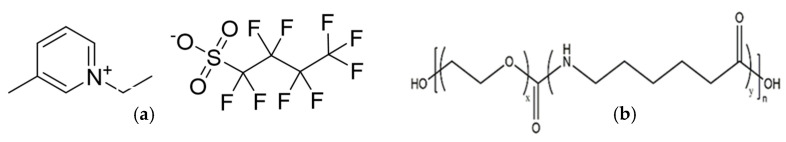
Structures of: (**a**) FIL used in this work, 1-ethyl-3-methylpyridinium perfluorobutanesulfonate ([C_2_C_1_py] [C_4_F_9_SO_3_]); and (**b**) molecular structure of repeated unit of Pebax^®^1657MH.

The nanosheets were supplied by XG Sciences, Inc. (Lansing, MI, USA), having a surface area of 750 mm^2^.g^−1^ and a thickness of 1–5 nm. The FIL used was 1-ethyl-3-methylpyridinium perfluorobutanesulfonate [C_2_C_1_py] [C_4_F_9_SO_3_] (>99% mass fraction purity) supplied by Iolitec (Heilbronn, Germany). The thermophysical profile of this FIL has been determined in previous works [22,23,24], and its chemical structure is presented in Figure 1a. The values of [C_2_C_1_py] [C_4_F_9_SO_3_] at 30 °C used in this work were: density 1.51 g·cm^−3^, viscosity 150.3 mPa·s, and molar volume 279.01 cm^3^·mol^−1^.

The dispersion in the base fluid was carried out with an ultrasound bath (Ultrasons-HD by Selecta, Barcelona, Spain), the stability of these samples was previously analyzed, and was determined that the ideal dispersion time is 1 h.

### 2.2. Materials for Membrane Preparation

Butan-1-ol (99.9 wt%), purchased from VWR, was used as solvent for the preparation of all dense films. The block copolymer poly(ether-block-amide) Pebax1657^®^ MH grade chemical structure presented in Figure 1b was kindly provided as pellets by Arkema Química S.A. (Madrid, Spain), which consists of soft and flexible polyether blocks (PEO) interlinked with hard and rigid polyamide 6 (PA6) segments. The composition of this copolymer is 60 wt% PEO, 40 wt% PA6 and it has a density of 1.14 g·cm^−3^.

The IL 1-ethyl-3-methylpyridinium perfluorobutanesulfonate [C_2_C_1_py] [C_4_F_9_SO_3_] was used for the preparation of the composite ionic liquid and polymer membranes (CILPMs) and the synthetized [C_2_C_1_py] [C_4_F_9_SO_3_]-based IoNF was employed for the preparation of the mixed-matrix membranes (MMMs). For membrane preparation, all reagents (solvent, polymer, IL and IoNF) were used as received without any further purification step.

For membrane preparation, near 3 wt% of polymer was dissolved in butan-1ol at 100 °C under magnetic stirring. Once the polymer was dissolved, the corresponding amount of either IL or IoNF was added and stirring was maintained at 100 °C for 1 h more to ensure homogenization and to prevent the gelation of the mixture. Afterwards, the solution was poured onto a glass Petri dish, and the solvent was evaporated into a vacuum oven at 300 mbar of absolute pressure and 40 °C overnight.

Membranes thickness was measured with a Mitutoyo digital micrometer (MDC-25PX, accuracy ± 1 μm, Neuss, Germany). An average value of 100 ± 10 μm was obtained from nine measurements at different points of the membrane.

### 2.3. Membrane Characterization

The prepared IoNFs and membranes were characterized by ATR-FTIR spectra (Nicolet 6700) in the range of 4000–400 cm^−1^ with a resolution of 4 cm^−1^ and 34 scans. The glass transition temperature (*T*_g_) of membranes was measured using differential scanning calorimeter (DSC) (Q2000 by TA Instruments, Spain) under a nitrogen atmosphere in the range of (−80) to 250 °C with a 10 °C/min scanning rate. Thermo-gravimetric analysis (TGA, Setsys Evolution 1750, Setaram) was utilized under a nitrogen atmosphere at a 10 °C/min ramp rate to evaluate membrane thermal stability of the membranes.

The morphology of membranes was observed by Scanning Electron Microscope (SEM), A FEI Quanta 200 environmental scanning electron microscope (ESEM). It was used to characterize the surface and the cross-sectional morphology of the samples that were fixed on an adhesive carbon tape and coated with gold. The cross-sections were prepared by cryogenic fracturing in liquid nitrogen. Secondary electron (SE) images were taken at an accelerating voltage of 12.5 kV FEI. The surface morphology of the membranes was also characterized using white light interferometry (WLOP), WYKO NT 1100. The mode used is white light vertical scanning interferometry, (VSI). For the preparation of the samples, the least deformed pieces of membrane are chosen and fixed on an opaque sample holder, to help the reflection of light. They are fixed at the ends with an adhesive; so that they are as flat as possible without becoming deformed.

## 3. Results and Discussion

In this work, a total of six IoNFs and nine functionalized polymeric membranes (one neat Pebax^®^1657 membrane, three CILPMs and five MMMs) were analyzed at different concentrations. The concentrations of copolymer, IL and graphene nanoparticles are shown in Table 1 for each IoNF and functionalized membranes studied in this work. The results obtained for all IoNFs and membranes are shown in the Supporting Information. In this section, only the most relevant results have been selected for discussion.

### 3.1. FTIR Characterization

**Figure 2:**
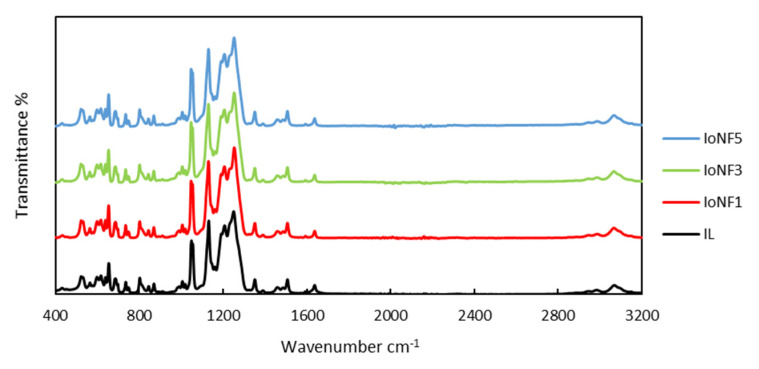
FTIR spectra of pure ionic liquid (IL) and of 3 IoNFs—full names in Table 1—with: 0.2 wt% xGnP (IoNF1); 2 wt% xGnP (IoNF3); and 8 wt% xGnP (IoNF5).

The FTIR spectra of IoNFs are shown in Figure 2. In practice, one can only see the characteristics peaks of the IL, [C_2_C_1_py] [C_4_F_9_SO_3_], in the IoNFs spectra, the bands of the graphene particles seem “invisible”. The spectrum of pristine graphene is well detailed in different works [25,26,27], and the prominent peaks are depicted in Table S1. The identification bands corresponding to the ionic liquid are reported in Table S2 [28,29]. The prominent groups in pristine graphene have a hydrophobic nature, determining the dispersibility behavior in aqueous and non-aqueous media, and mainly interact with the hydrophobic regions of the IL as detailed below. The bands between 2850 and 3000 cm^−1^ are due to the tension modes of the C–H bonds of CH_2_ and CH_3_ groups of the aliphatic chains attached to the pyridine ring. The bending modes appear between 1352 and 1459 cm^−1^, which increase slightly with the addition of graphene. The 3068 cm^−1^ band is due to the C–H bond tension modes of the aromatic ring of pyridine. The bands located between 1486 and 1637 cm^−1^ are due to the tension vibrations of the C=C and C=N bonds of the pyridine aromatic ring. The tension vibration modes of the C–F links of the CF_2_ and CF_3_ groups usually appear between 1000 and 1300 cm^−1^, while the bending modes appear between 500 and 850 cm^−1^, and all increase intensity with the addition of graphene. The bands between 1000 and 1070 cm^−1^ correspond to the symmetric tension mode of the SO_3_ group of the IL. The antisymmetric tension mode appears at a higher frequency, between 1100 and 1200 cm^−1^. The different bending modes of the SO_3_ group appear between 500 and 700 cm^−1^. These results show the good interaction between the IL with graphene even at high concentrations of graphene nanoparticles.

**Figure 3:**
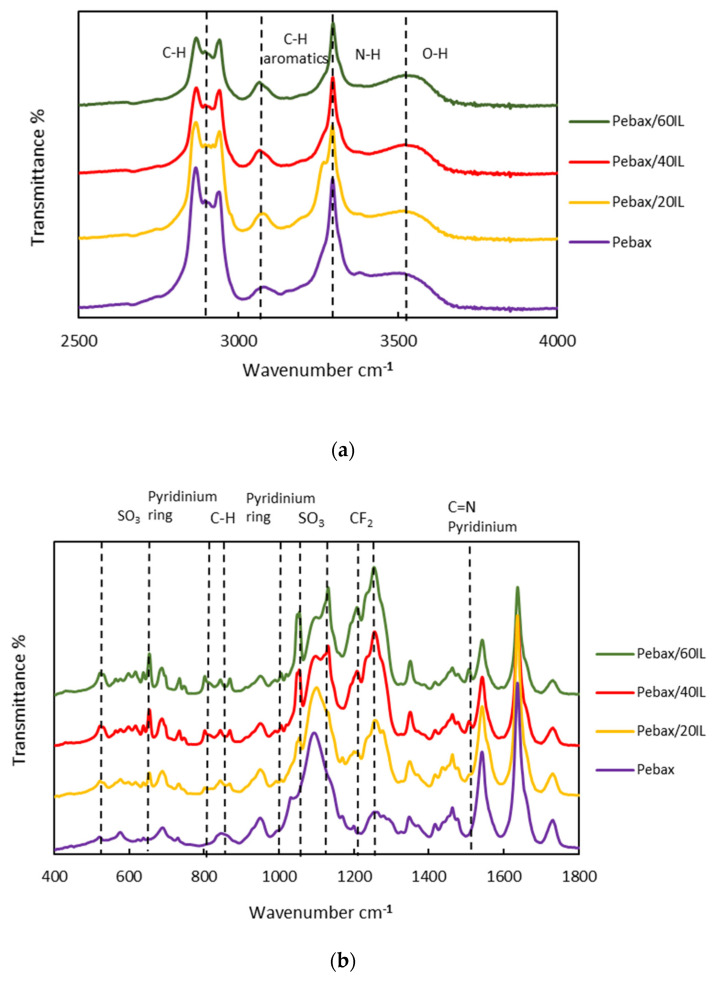
Comparison between the Pebax^®^1657 and the composite ionic liquid and polymer membranes (CILPMs) (Pebax/20IL, Pebax/40IL; Pebax/60IL—full names in Table 1) studied in this work in the range of: (**a**) 2500–4000 cm^−1^; (**b**) 1800–400 cm^−1^. [C_2_C_1_py] [C_4_F_9_SO_3_] characteristic peaks are identified.

Figure 3 illustrates the comparison of pure Pebax^®^1657 with the CILPMs prepared in this work. Figure S1 shows the comparison between CILPM Pebax/20IL and MMMs. The identification bands corresponding to the Pebax^®^1657 are shown in Table S3 [30]. Pebax^®^1657 characteristic peaks appear as stretching and bending vibrations of the NH group at 3297 and 1542 cm^−1^, respectively, the free peak of C=O at 1731 cm^−1^, C=O group in HNC=O at 1637 cm^−1^, and C–O–C bond stress peaks at 1094 cm^−1^ with the highest peak at 1090 cm^−1^. In Figure 3a, a slight change is observed, the 3500 cm^−1^ band moves slightly towards higher wave numbers, also a decrease in the intensity of the peaks and a slight narrowing are observed. In Figure 3b, it is observed that new bands appear in the CILPM samples, ~1600 cm^−1^ to pyridinium ring, ~1520 to C=N, ~1240 to CF_2_ and the R–SO_3_ group has several bending modes, one at 600–700 cm^−1^, which includes all the links in the group (S=O and SO) and another bending mode at 520–530 cm^−1^ that only has S=O bonds, that did not exist in pure Pebax^®^1657, and as the amount of IL increases, the intensity of these bands increases as well. The new, more intense bands are due to vibration modes of the CF_2_ and SO_3_ groups of the IL. Bands corresponding to the pyridinium ring of this compound also appear.

**Figure 4:**
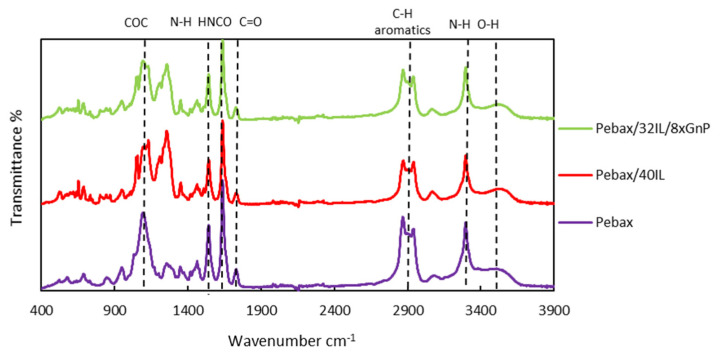
FTIR spectra of pure Pebax^®^1657, the CILPM Pebax/40IL and the mixed-matrix membrane (MMM) Pebax/32IL/8xGnP studied in this work—full names in Table 1. Pebax^®^1657 characteristic peaks are identified.

Figure 4 illustrates the FTIR spectra of the polymeric membranes: Pebax^®^1657, 60wt% Pebax^®^1657 + 40 wt% [C_2_C_1_py] [C_4_F_9_SO_3_] and 60 wt% Pebax^®^1657 + 32 wt%[C_2_C_1_py] [C_4_F_9_SO_3_] + 8 wt% xGnP. In addition, all characterized membranes are shown in Figure S2. The aforementioned Pebax^®^1657 characteristic peaks are detailed in Figure 4. The characteristic IL signal representing the C–H group on the cation ring appears as a peak between 2850–3000 cm^−1^, the peak being more intense than in pure Pebax^®^1657. Furthermore, the bending vibration of the IL cationic ring and the stretching peaks of ethyl-N and methyl-N can be found as broad peaks of 1046 and 1012 cm^−1^. When IL/xGnP was mixed with Pebax^®^1657, little change in the distribution of the polymeric membrane was observed. Furthermore, there were no additional peaks other than those of Pebax^®^1657, xGnP and IL in the mixture, confirming the absence of chemical reaction as a result of the IL/xGnP mixture. No significant peaks were observed for graphene, indicating the lack of functional groups xGnP-polymer.

### 3.2. Thermogravimetric Analysis

**Figure 5:**
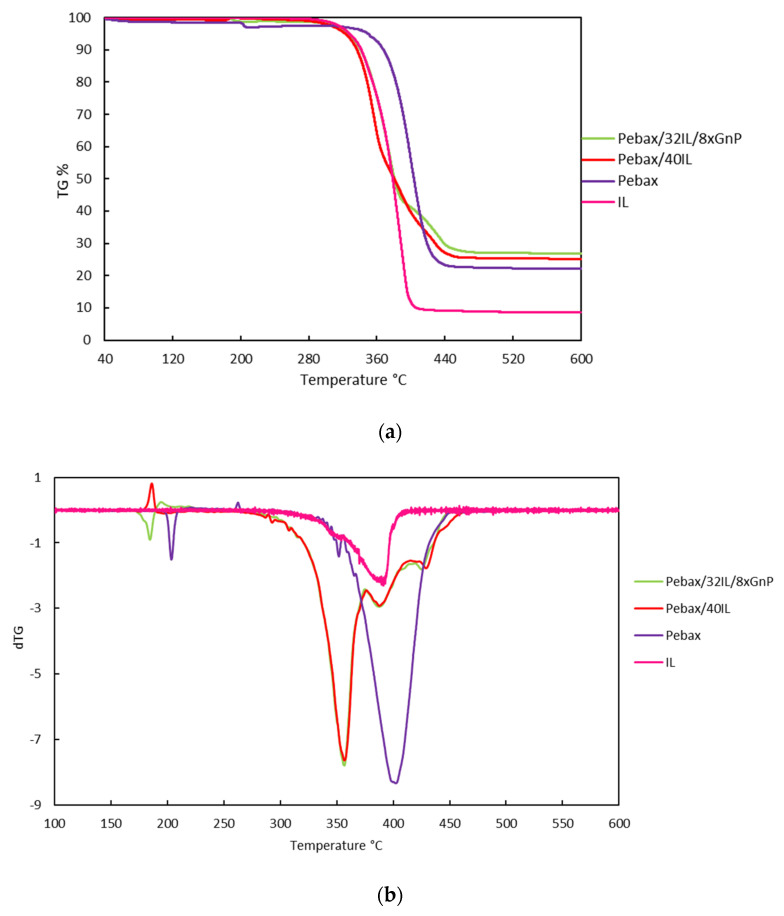
TGA curves (**a**) and dTGA curves (**b**) of IL, Pebax; Pebax/40IL; and Pebax/32IL/8xGnP—full names in Table 1.

Pebax-IoNFs degradation can be tested by the thermal stability of the membrane determined by TGA. Figure 5 shows the TGA and DTGA curves of the neat Pebax^®^1657 and the composites containing xGnP-IL fillers and the previously reported IL [22]. Figures S3–S5 show the TGA and DTGA curves for all characterized membranes. In the Figure 5, Pebax^®^1657 showed a two-stage decomposition, related to the random cleavage mechanism of the main polymer chain. The functionalized membranes showed a similar behavior to pure Pebax^®^1657. The onset (*T*_Onset_), starting (*T*_Start_) and decomposition (*T*_Dec_) temperatures, corresponding to the temperature at which the baseline slope changed during heating, the starting point and the final point of the degradation, respectively, are shown in Table 2.

Decomposition starts earlier in MMMs compared to pure polymer and CILMPs. The onset temperature of the pure polymer is higher than that of CILMPs and MMMs. There is a greater influence on the degradation temperatures in CILPM than in MMMs, which have similar values at different concentrations of graphene and ionic liquid, as can be seen in Figures S3 and S4. However, the increment of the IL concentration increases the stability of CILMPs but the increment of xGnP does not increase the stability of these membranes. The CILPM and MMM samples present three decomposition zones, presenting a new decomposition zone from 390 to 550 °C, with the exception of the 60% Pebax^®^1657 + 32% [C_2_C_1_py] [C_4_F_9_SO_3_] + 8% xGnP membrane, in which degradation occurs in a single step.

When the IoNF is incorporated into the Pebax^®^1657 polymer, the IoNFs penetrates between the Pebax^®^1657 chains. This reduced the thermal stability of the Pebax^®^1657/IoNFs composite membrane in comparison to pure copolymer. These results suggest that the Pebax^®^1657/IoNFs functionalized polymeric membrane may show enhanced separation properties as will be analyzed in-depth Part II of this study. As a result, thermal stability was reduced and degradation occurred at a lower temperature than pure Pebax^®^1657 [31,32] and the degradation profiles approximate the pure ionic liquid profile. Furthermore, CILPMs are more stable than MMMs. This behavior demonstrates that the transport properties and stability of this type of membranes is more influenced by the presence of IL than by graphene. However, the presence of graphene can play an important role in the selectivity of this type of membranes.

### 3.3. Thermal Analysis

**Figure 6:**
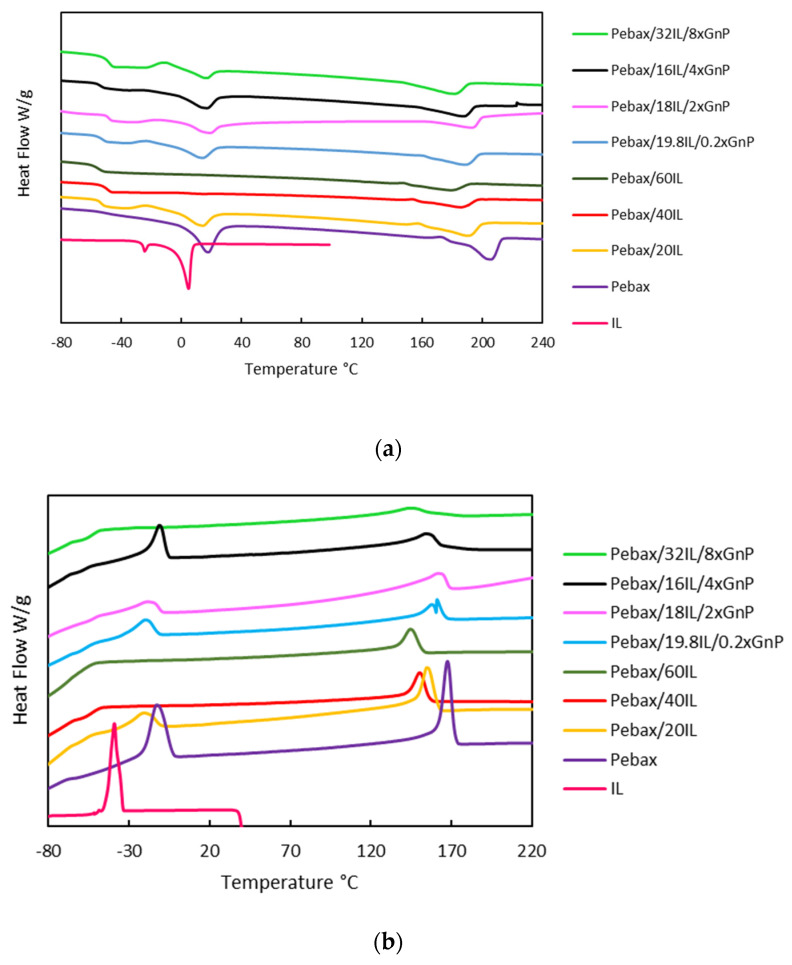
DSC thermograms of IL; Pebax; Pebax/20IL; Pebax/40IL; Pebax/60IL; Pebax/19.8IL/0.2 × GnP; Pebax/18IL/2 × GnP; Pebax/16IL/4 × GnP; and Pebax/32IL/8 × GnP at 10 °C/min: (**a**) heating ramp; and (**b**) cooling —full names in Table 1.

Heat flow measurements from thermograms were carried out to explore the effect of xGnP-IL fillers on the pristine Pebax^®^1657 sample. The results are shown in Figure 6, where the comparison between the DSC curve for the polymer Pebax^®^1657, for the pure IL [22,33] and the curves with the incorporation of IL and xGnP-IL are illustrated. Furthermore, Figure S6 illustrates the DSC curves for all characterized membranes and the results are reported in Table 3.

In the cooling thermogram, Figure 6a, the Pebax^®^1657 sample showed two exothermic peaks, which can be attributed to the crystalline fraction of poly(ethylene oxide) (PEO) and polyamide (PA6). On the other hand, crystallinity decreases with the addition of IL, as expected, [34] due to the steric hindrance presented by the ionic liquid. In addition, a complete disappearance of the second exothermic peak, except for the samples containing 80% of Pebax^®^1657. The MMMs show a clearly different behavior, as there is a shift in the positions of the first exothermic peak Pebax^®^1657. However, the second exothermic peak was maintained due to the xGnP presence, except for the highest xGnP-IL concentration where the exothermic peak at low temperatures disappears completely (the highest IL concentration).

In the heating thermogram, Figure 6b, two dominant endothermic peaks are present for neat Pebax^®^1657. The maxima of these peaks occur at 28 and 206 °C. These endotherms can be attributed to the melting of the crystalline fraction of the blocks of polyethylene oxide (PEO) and of polyamide (PA6), respectively [18,34,35]. The incorporation of IL produces a decrease in the peaks as a consequence of the decrease in the concentration of the Pebax^®^1657 polymer and a complete disappearance of the first endothermic peak, except for the samples containing 80% of Pebax^®^1657. With the addition of graphene, there is practically no change in the positions of the peaks and both crystalline phases remain present, which indicates little phase compatibility between the crystalline fraction of the polymer and the xGnP, in contrast to the high compatibility and good mixing of graphene and IL.

Therefore, the peak sizes decrease according to the decrease in the polymer concentration in the Pebax/IL mixture. The overall crystallinity is derived from the weight fraction of the two blocks and decreases rapidly with increasing IoNF content. In Pebax1657/IoNFs mixtures, the endothermic peak attributed to the PA6 crystalline fraction is shifted towards a lower temperature with an increasing content of IoNFs. The strong and relatively sharp peak of the PA6 blocks is replaced by a much weaker and broader melting peak, suggesting a reduction in the average crystal size and/or purity, while the PEO melting peak disappears for samples with high ionic liquid content in CILPMs, as seen in the literature [36]. Furthermore, this analysis showed that the enthalpy of fusion was in the range of 18–0.3 J/g for the PEO fraction and in the range of 22–10 J/g for the PA6 fraction, showing a decrease in the enthalpy of fusion of the polyether phase (PEO) with the increment of the IL content.

On the other hand, the effect of xGnP-IL fillers on the glass transition temperature (*T*_g_) of pristine Pebax^®^1657 was determined. The soft domains of PEO have a low *T*_g_ at −50 °C. The PA6 part of the Pebax^®^1657 shows a wide melting temperature starting at 160 °C while the *T*_g_ of the hard segment cannot be detected. Small changes in membrane *T*_g_ make it clear that xGnP-IL fillers could not provide strong interactions with PEO segments. However, the slope of *T*_g_ increases with the incorporation of xGnP-IL, indicating that the PEO chains suffer relatively little interference with the PA6 segments [37]. Furthermore, at higher xGnP loadings, the agglomeration of the xGnP sheets and the tendency of the particles to disperse in the PEO segments rather than the PA6 domains caused the *T*_g_ to shift to higher temperatures.

Thus, DSC provides an excellent tool for evaluating the miscibility of IL in Pebax^®^1657 copolymers, where the melting point depression and reduction in crystallinity is a measure of the strength of the interaction between the polymers and IL [38]. The absence of the IL melting peak in the composite is a strong evidence of miscibility of IL in Pebax copolymers. Summarizing, the present study evidences a high compatibility of IL and Pebax^®^1657 polymer.

### 3.4. Morphology

#### 3.4.1. Scanning Electron Microscope (SEM)

**Figure 7:**
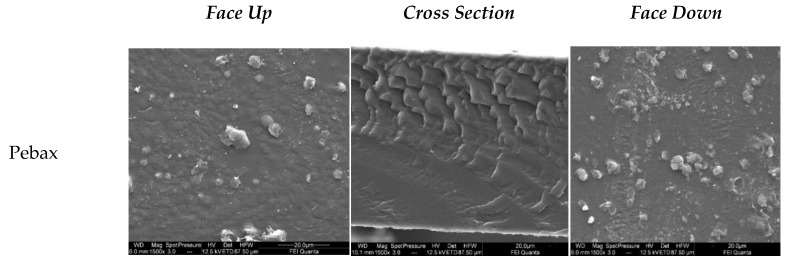

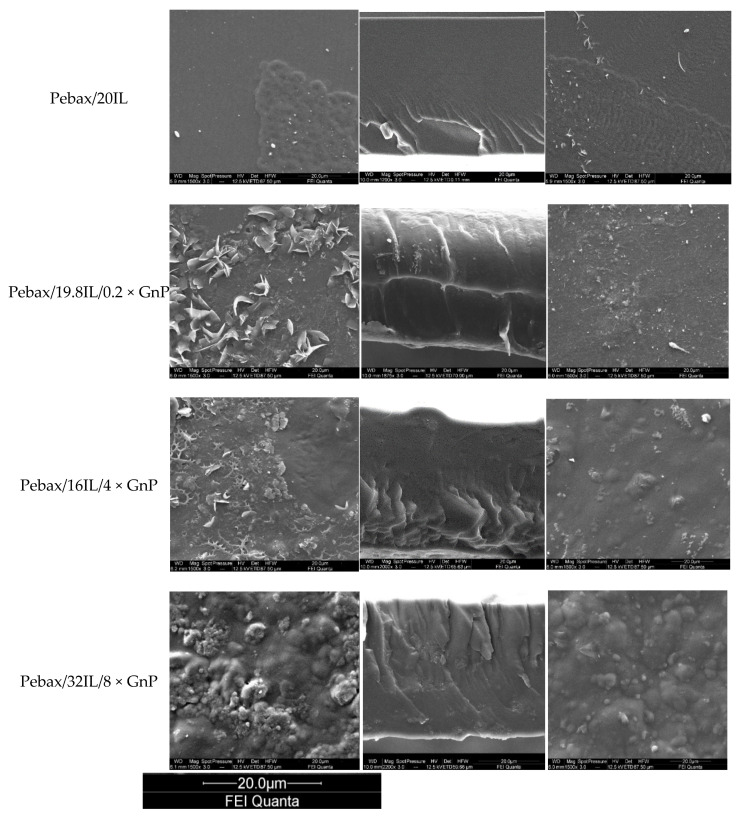
SEM images of Pebax; Pebax/20IL; Pebax/19.8IL/0.2 × GnP; Pebax/16IL/4 × GnP; and Pebax/32IL/8 × GnP—full names in Table 1.

SEM images of the surface and cross-sectional morphology of Pebax^®^1657, 80 wt% Pebax^®^1657 + 20 wt% [C_2_C_1_py] [C_4_F_9_SO_3_], 80 wt% Pebax^®^1657 + 19.8 wt% [C_2_C_1_py] [C_4_F_9_SO_3_] + 0.2 wt% xGnP, 80 wt% Pebax^®^1657 + 16 wt% [C_2_C_1_py] [C_4_F_9_SO_3_] + 4 wt% xGnP and 60 wt% Pebax^®^1657 + 32 wt% [C_2_C_1_py] [C_4_F_9_SO_3_] + 8 wt% xGnP are presented in Figure 7. Moreover, SEM images for all characterized membranes are shown in Figure S7. SEM analysis was also employed to investigate the dispersion and interfacial adhesion of IL and IL-xGnP within the Pebax^®^1657 matrix. The Pebax^®^1657 copolymer membrane shows a smooth and dense cross-sectional morphology [5]. However, by adding xGnP sheets, rough morphologies appeared on the surface of the membranes [37]. The face up images of 80 wt% Pebax^®^1657 + 19.8 wt% [C_2_C_1_py] [C_4_F_9_SO_3_] + 0.2 wt% xGnP, 80 wt% Pebax^®^1657 + 16 wt% [C_2_C_1_py] [C_4_F_9_SO_3_] + 4 wt% xGnP show that some of the xGnP laminates formed in the MMMs appear perpendicular to the surface of the polymeric membrane.

Agglomeration of xGnP was observed at the highest concentration values (8 wt% xGnP for Pebax/32IL/8xGnP and 12 wt% xGnP for Pebax/48IL/12xGnP, see Figure 7 and Figure S7). In addition, these membranes with higher IL content exhibit a homogeneous morphology revealing a uniform dispersion of IL-xGnP in the polymer matrix without aggregation or voids at the IoNFs/polymer interface—as can be seen in Figure 7 and Figure S7. The surface of the CILPMs membranes is smoother and the structure of the membranes becomes more amorphous with increasing IL content. This can be attributed to the reduction in crystallinity. These results justify that the modification of the surface with IoNFs improved the compatibility of the interface.

#### 3.4.2. Interferometric Optical Profilometry

A topographic and morphological study of the nine polymeric membranes was carried out using interferometric optical profilometry (WLOP) for each of their faces, since the membranes present roughness differences. Although WLOP has a lower resolution than atomic force microscopy (AFM), it provides measurements of larger fields of view. Then, a larger area can be analyzed, allowing the analysis to be more representative for this type of samples. Two measurement modes were used: (1) phase shift interferometry (PSI) mode which enables the high-resolution measurement of smooth surfaces and small steps; (2) vertical scan interferometry (VSI) mode which enables the measurement of rough surfaces and steps up to several millimeters in height.

**Figure 8:**
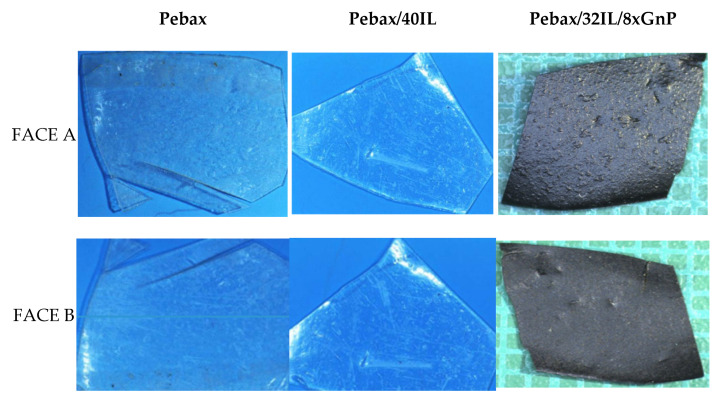
2D imaging with optical reflection (14.5 × 10.5 mm^2^) of rough face A and face B of Pebax, Pebax/40IL; and Pebax/32IL/8xGnP (full names in Table 1).

**Figure 9:**
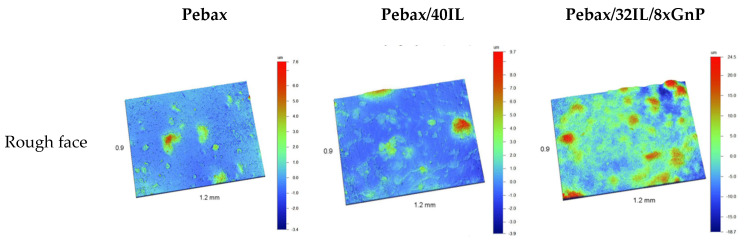

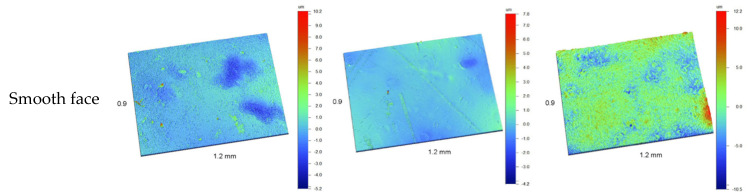
3D imaging with the topographic enhancement of the rough face and smooth face.

Each of the membranes has two faces with different topographic properties, one face being quite smooth and the other quite rough. Figure 8 and Figure S8 shows the optical images of both sides of the samples, optical images are qualitatively analyzed with a field of view of 14.5 × 10.5 mm^2^. In the most transparent samples, without graphene, it is difficult to differentiate roughness differences in optical reflection images. In the detailed results of interferometric profilometry, Figure 9 and Figure S9, we obtain significant topographic differences, allowing to classify the rough and smooth faces.

**Figure 10:**
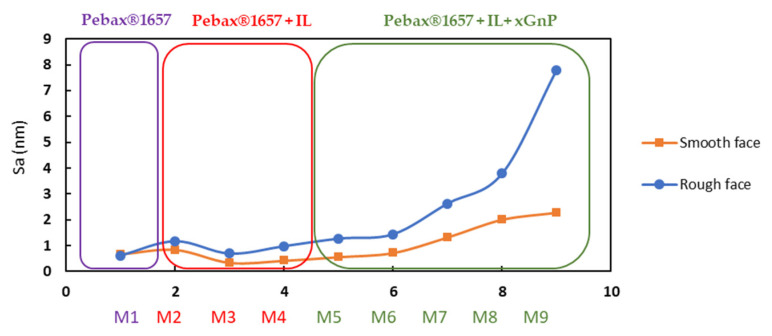
Trend of Sa parameter for the smooth and rough faces of Pebax (M1); Pebax/20IL (M2); Pebax/40IL (M3); Pebax/60IL (M4); Pebax/19.8IL/0.2 × GnP (M5); Pebax/18IL/2 × GnP (M6); Pebax/16IL/4 × GnP (M7); Pebax/32IL/8 × GnP (M8); and Pebax/48IL/12 × GnP (M9)—full names in Table 1.

The Pebax^®^1657 sample presents a similar roughness on both sides of the membrane Figure 9, it shows the same level of roughness and it is indistinct to classify one side or the other as smooth or rough. However, topographic differences are observed in Figure 10, although in the calculation of roughness, they do not produce appreciable differences, observing a frequency of peaks in the rough samples; that is, high areas, while in the smooth face; valleys are predominant.

The 5X objective of WLOP offers a field of view of 1.2 × 0.9 mm^2^. From these images, we calculated the 3D amplitude parameters that define the roughness to quantitatively assess the surface texture of the polymeric membranes. For the calculation of the three-dimensional roughness parameters (S_a_, S_q_, S_z_, S_sk_ and S_ku_), a “Cylinder and Tilt” correction was applied beforehand to eliminate plane inclination and minimize the influence of geometric deformations. The calculations were carried out without applying the interpolation in the few missing areas of pixels, to represent the images and improve their visual quality, and the “Data Restore” interpolation was applied. Amplitude parameters S_a_ and S_q_ are the mean roughness and root mean square (RMS) roughness evaluated over the complete 3D surface, respectively. Mathematically, S_a_ and S_q_ can be evaluated as follows:

(1) $$S_{a}\;=\int \int_{a}\left|Z\left(x,y\right)\right|dxdy$$

(2) $$S_{q}\;=\sqrt{\int \int_{a}{\left(Z\left(x,y\right)\right)}^{2}dxdy}$$

S_sk_ and S_ku_ are the skewness and kurtosis of the 3D surface texture, respectively, representing a histogram of the heights from an ideal normal distribution. Mathematically, S_sk_ and S_ku_ can be evaluated as follows:

(3) $$S_{sk}=\frac{1}{S_{q}^{3}}\int \int_{a}{\left(Z\left(x,y\right)\right)}^{3}dxdy$$

(4) $$S_{ku}=\frac{1}{S_{q}^{4}}\int \int_{a}{\left(Z\left(x,y\right)\right)}^{4}dxdy$$

S_sk_ represents the degree of symmetry of the surface heights about the mean plane. The sign of S_sk_ indicates the preponderance of peaks (that is, S_sk_ > 0) or valley structures (S_sk_ < 0) comprising the surface. S_sk_ is useful to characterize honed surfaces and wear measurements. On the other hand, S_ku_ indicates the nature of the height distribution. Therefore, it is useful to indicate the presence of either peak or valley defects, such as scratches. If the surface heights are normally distributed, then S_ku_ is 3.00. Surfaces composed of inordinately high peaks/deep valleys have S_ku_ > 3.00. S_ku_ indicates gradually varying surfaces.

S_p_, S_v_, and S_z_ are the parameters evaluated from the absolute highest and lowest points found on the surface. S_p_, the maximum peak height, is the height of the highest point, S_v_, the maximum valley depth, is the depth of the lowest point (expressed as a negative number) and S_z_, the maximum height of the surface, is calculated from S_z_ = S_p_–S_v_.

The results obtained for the membranes studied in this work are reported in Table 4 and S_a_ parameter is plotted in Figure 10.

On the smooth side, some differences are observed in the roughness values, although they are less noticeable than on the rough side. The smooth faces have been in contact with a relatively flat support or surface during their synthesis which explain the behavior observed in this work. There is a coincidence in the tendency of the roughness of both the smooth and rough faces, the rough face being more pronounced. On the rough side, although an increase in roughness is observed, Pebax^®^1657 and the three CILPMs membranes present low roughness values and close to each other. The incorporation of graphene in the membrane clearly increases the three-dimensional roughness parameters.

## 4. Conclusions

As described above, graphene and ionic liquid-functionalized polymeric membranes have received much attention for gas separation applications and have seen remarkable progress in recent years. The search for surface modifications that generate compatibility between polymers and their aggregates, suppressing the plasticization of polymers and taking advantage of the synergistic effects, can improve the sustainability of these membranes towards gas capture applications. In this first paper, a series of functionalized polymeric MMMs were prepared by incorporating IL and graphene (graphene ionanofluids) into the Pebax^®^1657 matrix. The membranes obtained were characterized by FTIR, TGA, DSC, SEM and WLOP. The goal of the incorporation of surfactant IL and graphene is to improve the permeability and selectivity of these novel materials. This work demonstrated the high compatibility of IoNFs with the copolymer Pebax^®^1657 and these novel MMMs can be prepared using a simple solution molding technique.

The incorporation of IoNF in the Pebax^®^1657 matrix does not change the FTIR spectrum of the Pebax^®^1657 polymer. In addition, there are no additional peaks, confirming the absence of chemical reaction. When the IoNF is incorporated into the Pebax^®^1657 polymer, it penetrates between the Pebax^®^1657 chains, increasing the free volume of the polymer chain. This reduces the thermal stability of the Pebax^®^1657/IoNFs composite membrane. These results suggest that the Pebax^®^1657/IoNFs functionalized polymeric membrane showed improved permeability due to the high free volume of the polymer chain in it. In the DSC analysis, a great depression of the melting point of the PEO blocks is observed when the concentration of IL increases in the mixtures. This finding indicates a strong and favorable interaction between IL and PEO. In addition, this decrease in crystallinity leads to improved gas transport properties.

SEM analysis showed that the Pebax^®^1657 copolymer membrane presents a smooth and dense cross-sectional morphology. An amorphous structure appears with the addition and increment of the IL concentration, and the addition of xGnP sheets produces rough morphologies on the surface of the membranes, forming an important proportion of laminates in the MMMs oriented perpendicularly to the surface of the polymeric membrane. Finally, the morphological and topographic study was used to determine the three-dimensional roughness parameters that also demonstrated the increment of the roughness with the incorporation of graphene in the membranes. 

Polymers are generally used to make membranes due to their excellent gas separation properties. However, they have a comprehensive compromise between permeability and selectivity, and sensitivity to high temperatures, high pressures and harsh chemical environments. For this reason, it is important to perform a comprehensive study of their characterization, evaluating in this particular case the effect of the ionic liquid and graphene addition on the microstructure of these membranes.

## Figures and Tables

**Table 1:** Abbreviated name and full name (description of the membranes and ionanofluids (IoNFs)) studied in this work.

Abbreviated Name of Membranes		Full Name (Mass Compositions of Polymer, Ionic Liquid, IL, and Graphene, xGnP)
Pebax	M1	Pebax^®^1657
Pebax/20IL	M2	80 wt% Pebax^®^1657 + 20 wt% [C_2_C_1_py] [C_4_F_9_SO_3_]
Pebax/40IL	M3	60 wt% Pebax^®^1657 + 40 wt% [C_2_C_1_py] [C_4_F_9_SO_3_]
Pebax/60IL	M4	40 wt% Pebax^®^1657 + 60 wt% [C_2_C_1_py] [C_4_F_9_SO_3_]
Pebax/19.8IL/0.2xGnP	M5	80 wt% Pebax^®^1657 + 19.8 wt% [C_2_C_1_py] [C_4_F_9_SO_3_] + 0.2 wt% xGnP
Pebax/18IL/2xGnP	M6	80 wt% Pebax^®^1657 + 18 wt% [C_2_C_1_py] [C_4_F_9_SO_3_] + 2 wt% xGnP
Pebax/16IL/4xGnP	M7	80 wt% Pebax^®^1657 + 16 wt%[C_2_C_1_py] [C_4_F_9_SO_3_] + 4 wt% xGnP
Pebax/32IL/8xGnP	M8	60 wt% Pebax^®^1657 + 32 wt%[C_2_C_1_py] [C_4_F_9_SO_3_] + 8 wt% xGnP
Pebax/48IL/12xGnP	M9	40 wt% Pebax^®^1657 + 48 wt%[C_2_C_1_py] [C_4_F_9_SO_3_] + 12 wt% xGnP

**Table 2:** Thermal properties obtained by TGA, temperature at which degradation begins; *T*_Start_, temperature at which degradation ends; *T*_Dec_, and the onset temperature; *T*_Onset_, of the different membranes.

	*T*_Start_ (°C)	*T*_Dec_ (°C)	*T*_Onset_ (°C)	*T*_Start_ (°C)	*T*_Dec_ (°C)	*T*_Onset_ (°C)	*T*_Start_ (°C)	*T*_Dec_ (°C)	*T*_Onset_ (°C)
	*1 Degradation Step*			*2 Degradation Step*			*3 Degradation Step*		
Pebax	179	238	204	277	486	399	−	−	−
Pebax/20IL	160	225	191	254	395	372	394	546	419
Pebax/40IL	242	377	358	375	416	387	413	550	429
Pebax/60IL	255	375	352	375	484	406	−	−	−
Pebax/19.8IL/0.2 × GnP	162	224	192	250	397	375	397	511	428
Pebax/18IL/2 × GnP	180	216	194	259	395	373	395	489	420
Pebax/16IL/4 × GnP	170	242	192	259	402	367	402	480	424
Pebax/32IL/8 × GnP	−	−	−	230	461	352	−	−	−

**Table 3:** Crystallization temperatures, *T*_crys_; melting temperatures, *T*_m_; and enthalpy, Δ*H*, at 10 °C/min.

	Cooling					Heating			
	*T*_crys_ 1 (°C)	Enthalpy 1 (J/g)	*T*_crys_ 2 (°C)	Enthalpy 2 (J/g)	*T*_m_ 1 (°C)	Enthalpy 1 (J/g)	*T*_m_ 2 (°C)	Enthalpy 2 (J/g)	*T*g (°C)
Pebax	−13	22.12	168	25.47	28	18	206	22.07	−50
Pebax/20IL	−21	9.26	155	18.31	14	16	190	16.11	−54
Pebax/40IL	−	−	150	12.50	−	−	186	9.84	−49
Pebax/60IL	−	−	144	12.94	−	−	149	9.19	−55
Pebax/19.8IL/0.2xGnP	−20	9.82	161	8.21	13	14.95	189	13.40	−52
Pebax/18IL/2xGnP	−19	12.72	162	14.25	18	18.53	193	11.77	−50
Pebax/16IL/4xGnP	−11	12.57	154	13.87	16	15.46	187	12.83	−54
Pebax/32IL/8xGnP	−	−	154	15.07	12	0.33	184	9.63	−49

**Table 4:** Interferometric optical profilometry of the (a) rough and (b) smooth faces of the membranes studied.

**(a) Interferometric Optical Profilometry/3D Amplitude Roughness Parameters/1.2 × 0.9 µm^2^ Field of View/ROUGH FACES**					
	**S_a_ (nm)**	**S_q_ (nm)**	**S_z_ (nm)**	**S_sk_**	**S_ku_**
Pebax	0.62	0.98	15.31	0.94	13.43
Pebax/20IL	1.17	1.65	16.58	1.44	7.49
Pebax/40IL	0.69	1.12	14.65	3.35	20.71
Pebax/60IL	0.98	1.34	15.35	0.02	5.10
Pebax/19.8IL/0.2 × GnP	1.27	1.82	14.19	1.94	9.85
Pebax/18IL/2 × GnP	1.44	2.02	21.72	1.62	11.48
Pebax/16IL/4 × GnP	2.63	3.63	32.43	1.49	8.78
Pebax/32IL/8 × GnP	3.79	5.26	40.17	1.385	7.08
Pebax/48IL/12 × GnP	7.80	10.21	56.08	0.80	5.39
**(b) Interferometric Optical Profilometry/3D Amplitude Roughness Parameters/1.2** × **0.9 µm^2^ Field of View/SMOOTH FACES**					
	**S_a_ (nm)**	**S_q_ (nm)**	**S_z_ (nm)**	**S_sk_**	**S_ku_**
Pebax	0.66	0.95	13.99	0.06	8.58
Pebax/20IL	0.83	1.12	12.20	−0.14	5.28
Pebax/40IL	0.33	0.46	8.97	0.39	15.60
Pebax/60IL	0.41	0.73	12.00	−5.03	67.22
Pebax/19.8IL/0.2 × GnP	0.55	0.74	7.31	−0.46	5.59
Pebax/18IL/2 × GnP	0.71	1.00	13.71	0.20	7.19
Pebax/16IL/4 × GnP	1.31	1.70	16.20	0.25	5.79
Pebax/32IL/8 × GnP	1.99	2.59	19.84	0.27	4.33
Pebax/48IL/12 × GnP	2.26	2.85	19.95	−0.12	3.63

## Data Availability

The data presented in this study are available on request from the corresponding author.

## Supplementary Materials

The following are available online at https://www.mdpi.com/2079-4991/11/3/607/s1, Figure S1. Comparison between CILPM Pebax/20IL and MMMs (full names in Table 1) with differences concentrations of IL and xGnP: a) range 2500-4000cm-1, b) range 400-2400cm-1, Figure S2. FTIR spectra of Pebax, Pebax/20IL, Pebax/40IL, Pebax/60IL, Pebax/19.8/IL/0.2xGnP, Pebax/18IL/2xGnP, Pebax/16IL/4xGnP, Pebax/32/8xGnP, and Pebax/48IL/12xGnP (full names in Table 1), Figure S3. TGA Curves of IL, Pebax, Pebax/20IL, Pebax/40IL, Pebax/60IL, Pebax/19.8/IL/0.2xGnP, Pebax/18IL/2xGnP, Pebax/16IL/4xGnP, and Pebax/32/8xGnP (full names in Table 1), Figure S4. TGA Curves of a) CILPMs: Pebax/20IL, Pebax/40IL, and Pebax/60IL; and b) MMMs: Pebax/19.8/IL/0.2xGnP, Pebax/18IL/2xGnP, Pebax/16IL/4xGnP, and Pebax/32/8xGnP (full names in Table 1), Figure S5. dTGA Curves of Pebax, Pebax/20IL, Pebax/40IL, Pebax/60IL, Pebax/19.8/IL/0.2xGnP, Pebax/18IL/2xGnP, Pebax/16IL/4xGnP, and Pebax/32/8xGnP (full names in Table 1), Figure S6. DSC Termograms of IL, Pebax, Pebax/20IL, Pebax/40IL, Pebax/60IL, Pebax/19.8/IL/0.2xGnP, Pebax/18IL/2xGnP, Pebax/16IL/4xGnP, and Pebax/32/8xGnP at 10°C/min: (a) cooling ramp, (b) heating ramp, Figure S7. STEM images of Pebax, Pebax/20IL, Pebax/40IL, Pebax/60IL, Pebax/19.8IL/0.2xGnP, Pebax/18IL/2xGnP, Pebax/16IL/4xGnP, Pebax/32IL/8xGnP, and Pebax/48IL/12xGnP (full names in Table 1), Figure S8. 2D imaging with optical reflection of Rough face A and face B of Pebax, Pebax/20IL, Pebax/40IL, Pebax/60IL, Pebax/19.8IL/0.2xGnP, Pebax/18IL/2xGnP, Pebax/16IL/4xGnP, Pebax/32IL/8xGnP, and Pebax/48IL/12xGnP (full names in Table 1), Figure S9. 3D imaging with topographic enhancement of Rough face and Smooth face of Pebax, Pebax/20IL, Pebax/40IL, Pebax/60IL, Pebax/19.8IL/0.2xGnP, Pebax/18IL/2xGnP, Pebax/16IL/4xGnP, Pebax/32IL/8xGnP, and Pebax/48IL/12xGnP (full names in Table 1). Table S1. Identification of bands corresponding to the graphene [S1-S3], Table S2. Identification of bands corresponding to the ionic liquid [S4,S5], Table S3. Identification of bands corre-sponding to Pebax®1657 [S6].

## Abbreviations

IoNF1	0.2 wt% xGnP/[C_2_C_1_py] [C_4_F_9_SO_3_]
IoNF2	0.4 wt% xGnP/[C_2_C_1_py] [C_4_F_9_SO_3_]
IoNF3	2 wt% xGnP/[C_2_C_1_py] [C_4_F_9_SO_3_]
IoNF4	4 wt% xGnP/[C_2_C_1_py] [C_4_F_9_SO_3_]
IoNF5	8 wt% xGnP/[C_2_C_1_py] [C_4_F_9_SO_3_]
IoNF6	12 wt% xGnP/[C_2_C_1_py] [C_4_F_9_SO_3_]
