# Supplementary material for: Integration of Stable Ionic Liquid-Based Nanofluids into Polymer Membranes. Part I: Membrane Synthesis and Characterization
Source: Nanomaterials (Basel). 2021 Feb 28;11(3):607. doi: 10.3390/nano11030607 (PMC7997425; doi:10.3390/nano11030607)
Supplement: Supplementary file 1 [file nanomaterials-11-00607-s001.pdf]

Supplementary Materials

Integration of Stable Ionic Liquid-Based Nanofluids into Polymer Membranes. Part I: Membrane Synthesis and Characterization

Carolina Hermida-Merino [1], Fernando Pardo [2], Gabriel Zarca [2], João M. M. Araújo [3], Ane Urtiaga [2], Manuel M. Piñeiro [1] and Ana B. Pereiro [3,*]

[1] Centro de Investigaciones Biomédicas (CINBIO), Department of Applied Physics, University of Vigo, E36310, Vigo, Spain; cahermida@uvigo.es (C.H.-M.) and mmpineiro@uvigo.es (M.M.P.)
[2] Department of Chemical and Biomolecular Engineering, Universidad de Cantabria, 39005 Santander, Spain; pardof@unican.es (F.P.); zarcag@unican.es (G.Z.); urtiaga@unican.es (A.U.)
[3] LAQV, REQUIMTE, Departamento de Química, Faculdade de Ciências e Tecnologia, Universidade Nova de Lisboa, 2829-516 Caparica, Portugal; jmmda@fct.unl.pt
* Correspondence: anab@fct.unl.pt; Tel.: (+351)-212-948-318

Table S1. Identification of bands corresponding to the graphene [S1-S3].

Description	Wavenumber (cm^{-1})
$=CH_2$ asymmetric and symmetric stretching	2915 and 2850
C-H bond stretching and bending	3783 and 672
C=C sp^2 bonds	1618
Adsorption of CO_2 from air	2300

Table S2. Identification of bands corresponding to the ionic liquid [S4,S5].

Description	Wavenumber (cm^{-1})
C-H bond tension	2850-3000
Pyridinium ring vibration	1600-1650
C=N bond tension, Pyridinium ring	1520-1450
CF_2 tension group	1240-1260
CF_2 tension group	1130
SO_3 tension group	1055, 1035, 1020
Pyridinium ring tension	1000-1030
SO_3 flexion group	600-700
O=S=O flexion of SO_3 group	520-530

Table S3. Identification of bands corresponding to Pebax®1657 [S6].

Composition	N-H bond tension	O-H bonds tension	C-H bonds tension	C=O bonds tension	HNCO tension	N-H flexion	C-O-C bond tension
Pebax	3296.5	3506	2850-3000	1731	1637	1542	1094
Pebax/20IL	3295.7	3520	2850-3000	1731	1637	1543	1101
Pebax/40IL	3297	3523	2850-3000	1731	1637	1543	1098
Pebax/60IL	3298	3520	2850-3000	1731	1638	1543	1099
Pebax/19.8IL/0.2xGnP	3296	3520	2850-3000	1731	1637	1542	1100
Pebax/18IL/0.2xGnP	3296	3519	2850-3000	1731	1637	1542	1097
Pebax/16IL/4xGnP	3296	3520	2850-3000	1731	1637	1542	1094
Pebax/32IL/8xGnP	3296.5	3521	2850-3000	1731	1637	1542	1094
Pebax/48IL/12xGnP	3296.5	3520	2850-3000	1731	1637	1543	1097

[1] Values in cm^{-1}

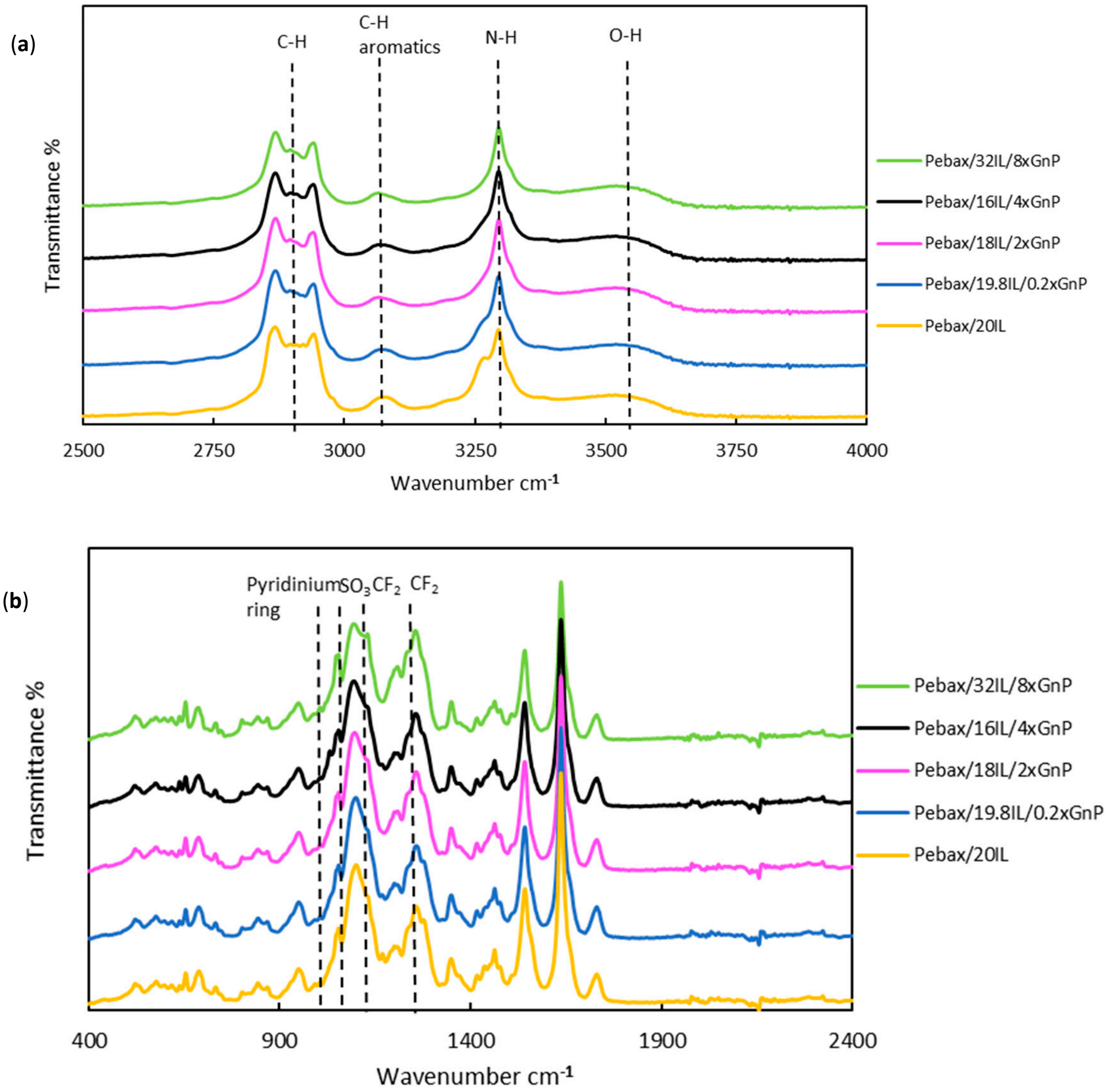

Figure S1. Comparison between CILPM Pebax/20IL and MMMs (full names in Table 1) with differences concentrations of IL and xGnP: a) range 2500-4000cm^{-1}, b) range 400-2400cm^{-1}.

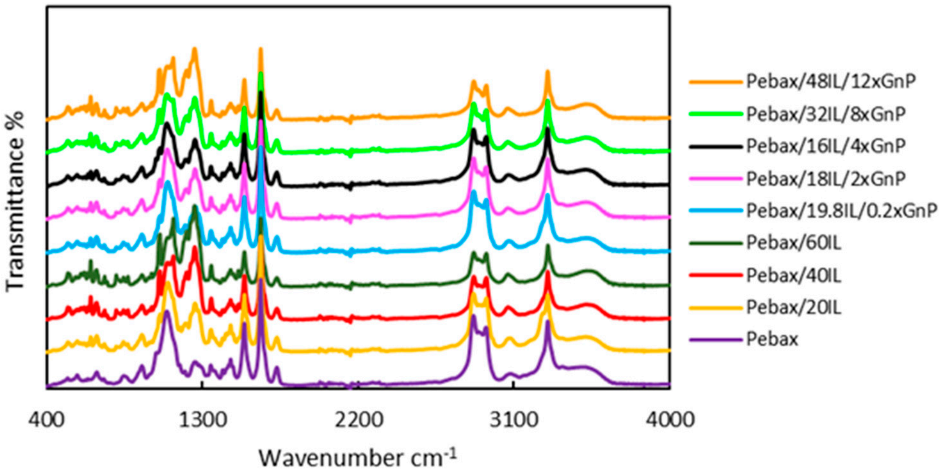

Figure S2. FTIR spectra of Pebax, Pebax/20IL, Pebax/40IL, Pebax/60IL, Pebax/19.8/IL/0.2xGnP, Pebax/18IL/2xGnP, Pebax/16IL/4xGnP, Pebax/32/8xGnP, and Pebax/48IL/12xGnP (full names in Table 1)

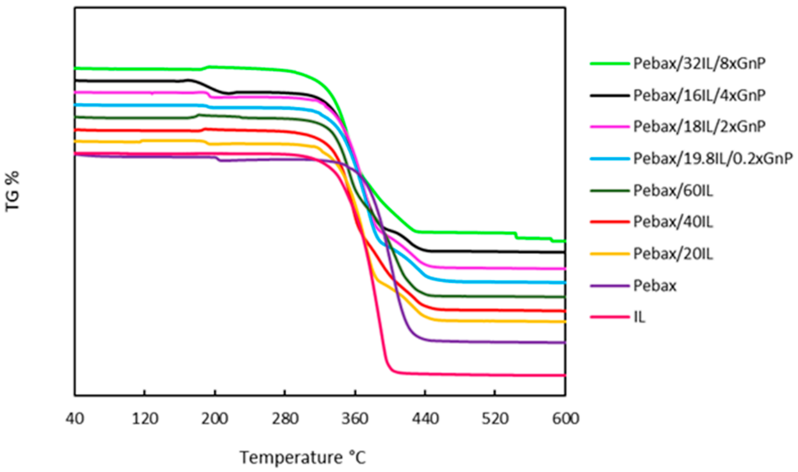

Figure S3. TGA Curves of IL, Pebax, Pebax/20IL, Pebax/40IL, Pebax/60IL, Pebax/19.8/IL/0.2xGnP, Pebax/18IL/2xGnP, Pebax/16IL/4xGnP, and Pebax/32/8xGnP (full names in Table 1).

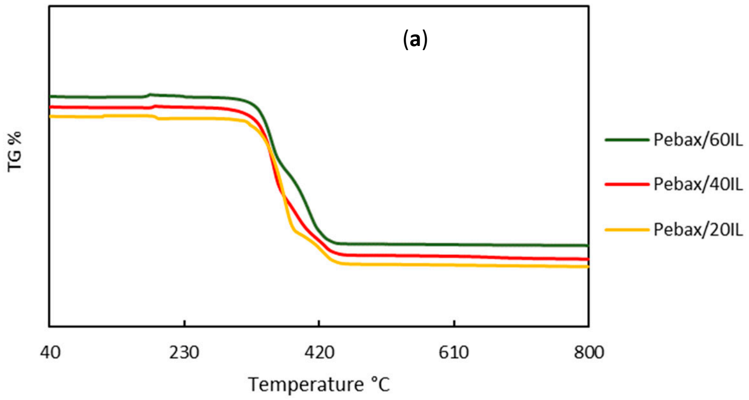

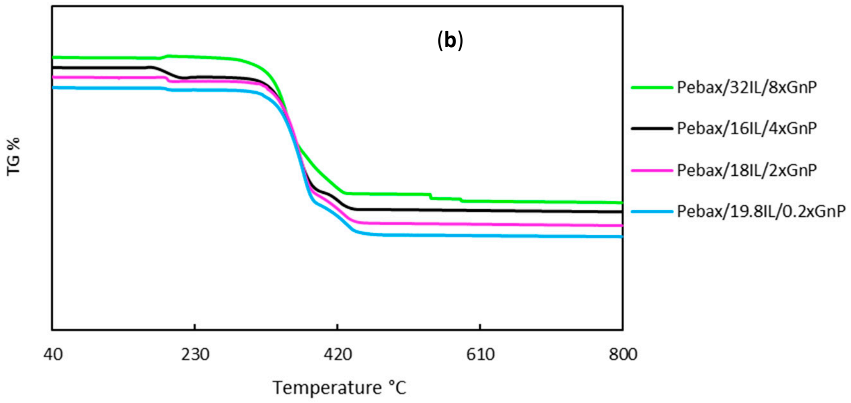

Figure S4. TGA Curves of a) CILPMs: Pebax/20IL, Pebax/40IL, and Pebax/60IL; and b) MMMs: Pebax/19.8/IL/0.2xGnP, Pebax/18IL/2xGnP, Pebax/16IL/4xGnP, and Pebax/32/8xGnP (full names in Table 1).

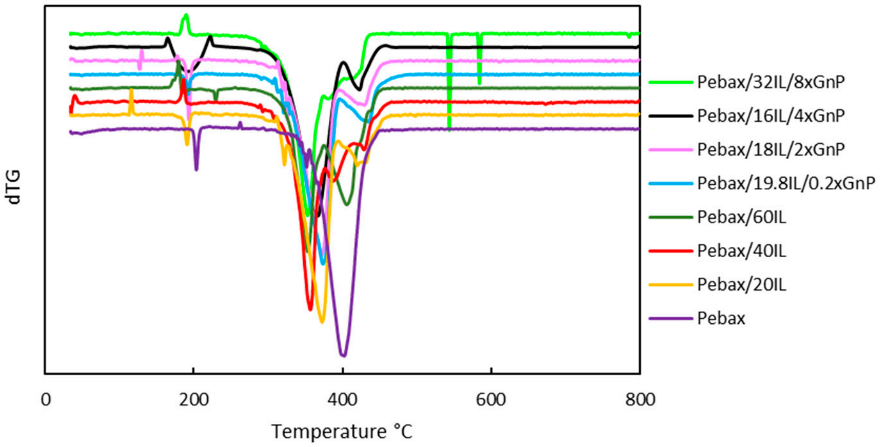

Figure S5. dTGA Curves of Pebax, Pebax/20IL, Pebax/40IL, Pebax/60IL, Pebax/19.8/IL/0.2xGnP, Pebax/18IL/2xGnP, Pebax/16IL/4xGnP, and Pebax/32/8xGnP (full names in Table 1).

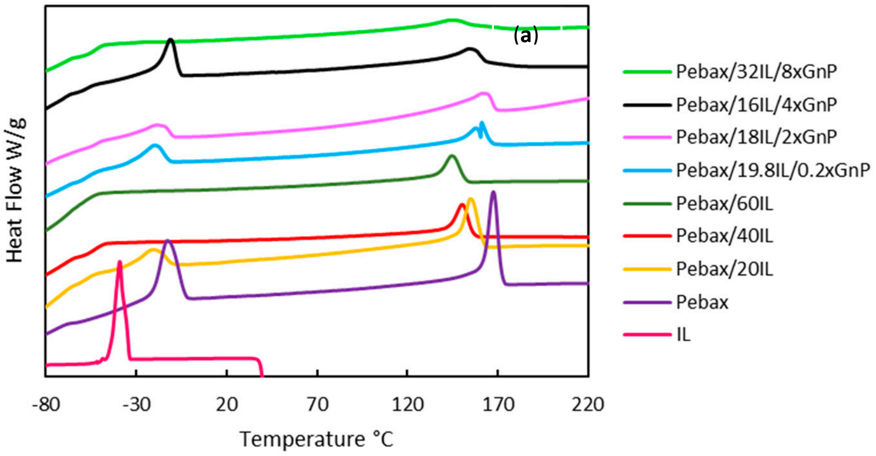

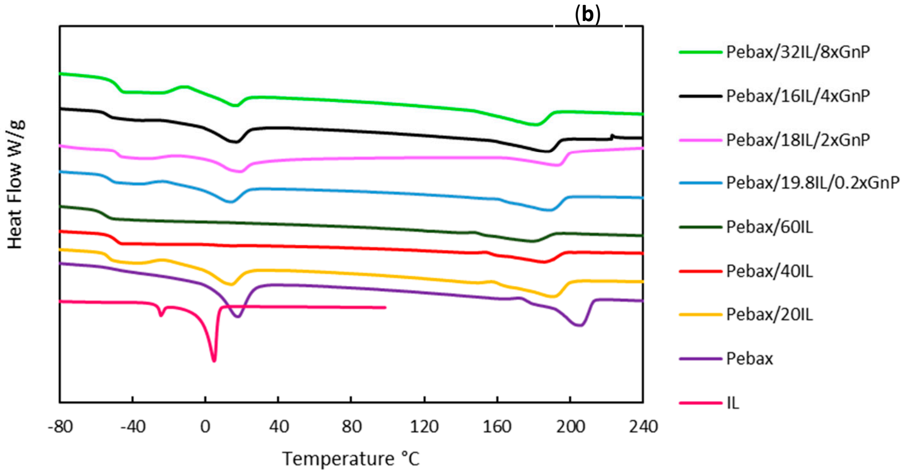

Figure S6. DSC Termograms of IL, Pebax, Pebax/20IL, Pebax/40IL, Pebax/60IL, Pebax/19.8/IL/0.2xGnP, Pebax/18IL/2xGnP, Pebax/16IL/4xGnP, and Pebax/32/8xGnP at 10°C/min: (a) cooling ramp, (b) heating ramp.

	Face up	*Cross Section*	*Face Down*
		Pure Pebax	
Pebax	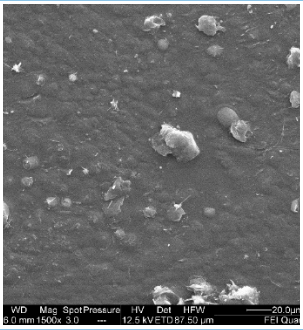	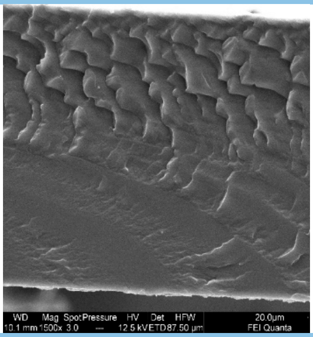	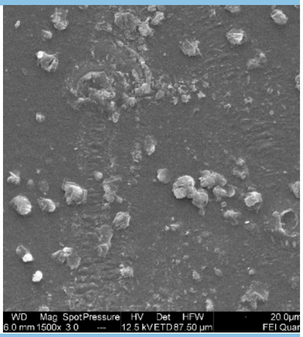
		Three CILPMs	
Pebax/20IL	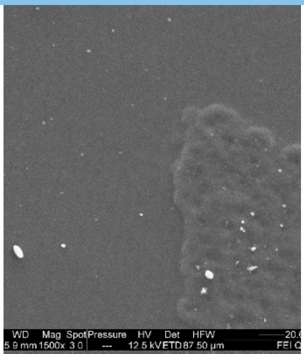	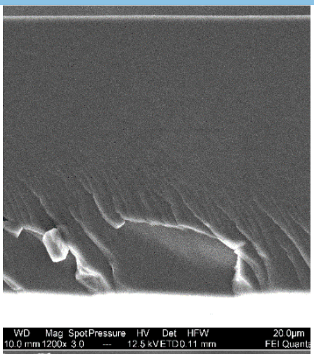	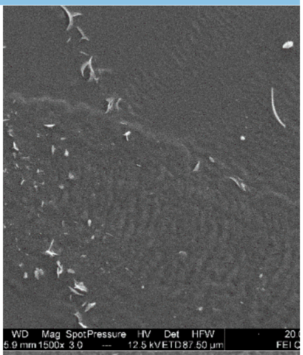
Pebax/40IL	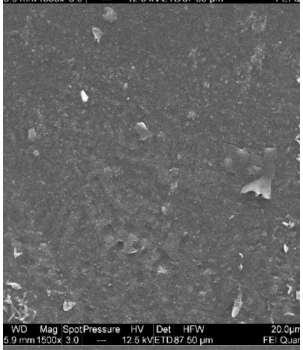	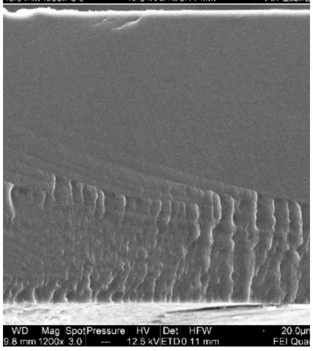	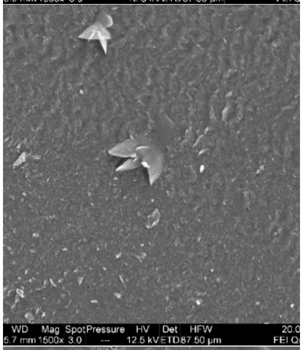
Pebax/60IL	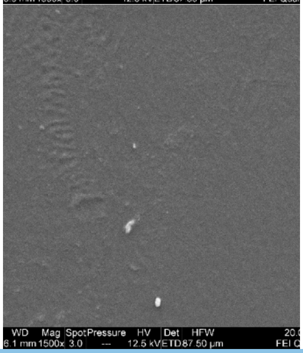	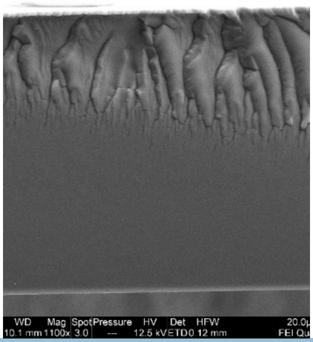	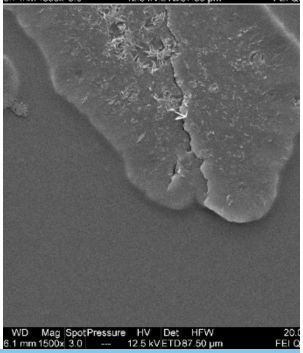
		Five MMMs	
Pebax/19.8IL/ 0.2xGnP	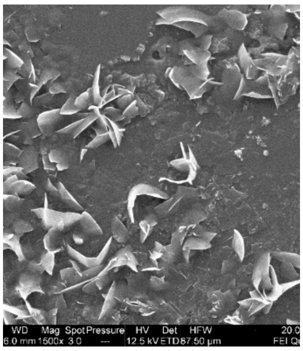	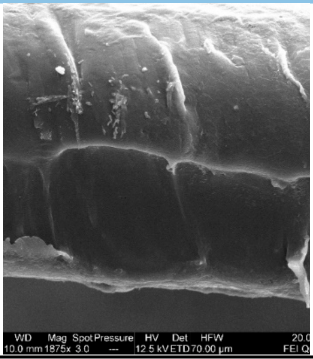	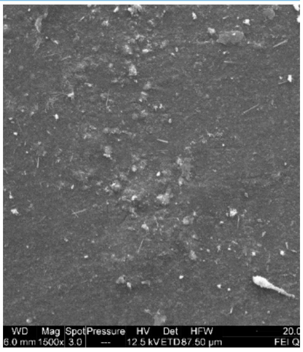

Pebax/18IL/
2xGnP

Pebax/16IL/
4xGnP

Pebax/32IL/
8xGnP

Pebax/48IL/
12xGnP

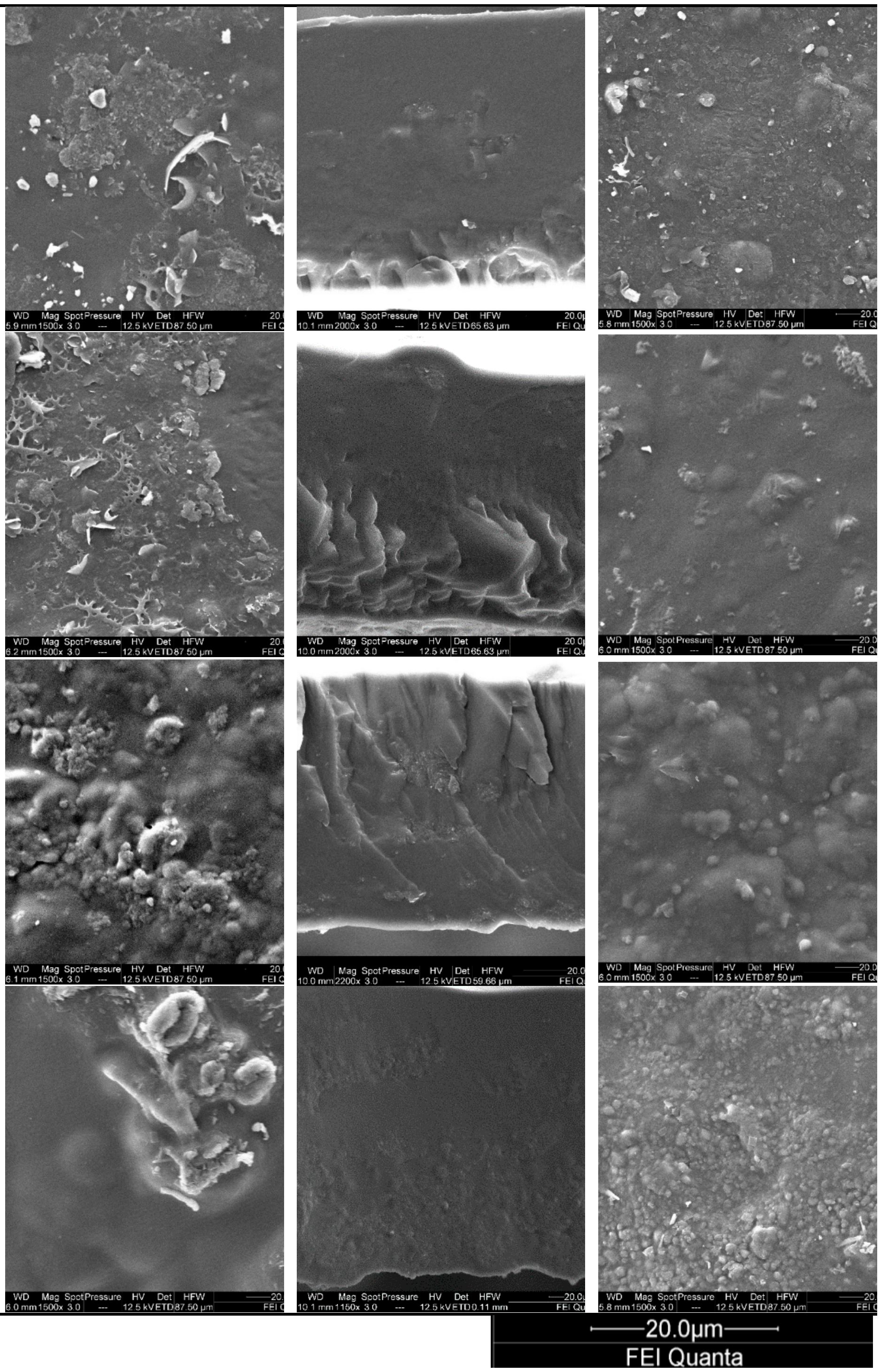

Figure S7. STEM images of Pebax, Pebax/20IL, Pebax/40IL, Pebax/60IL, Pebax/19.8IL/0.2xGnP, Pebax/18IL/2xGnP, Pebax/16IL/4xGnP, Pebax/32IL/8xGnP, and Pebax/48IL/12xGnP (full names in Table 1).

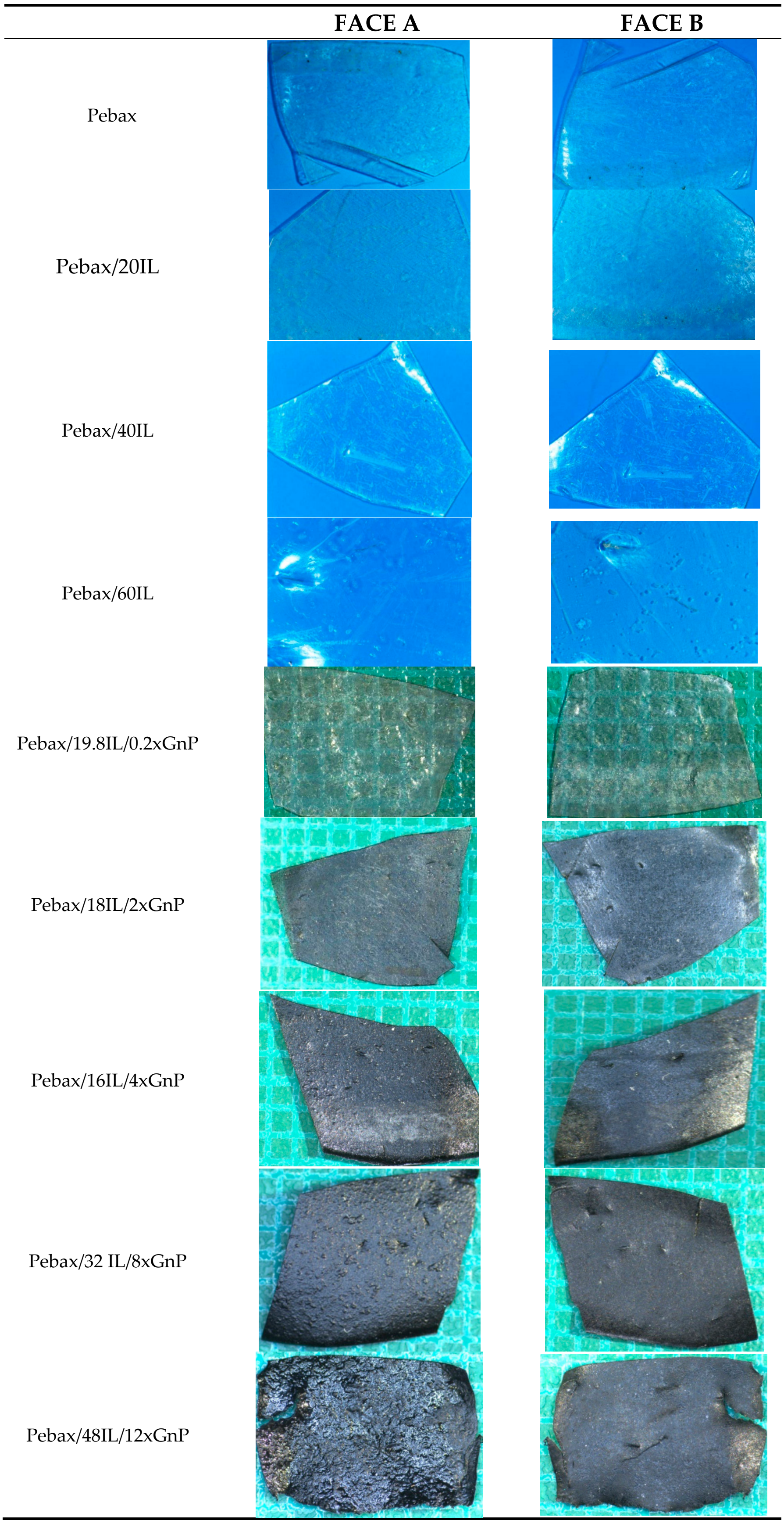

Figure S8. 2D imaging with optical reflection of Rough face A and face B of Pebax, Pebax/20IL, Pebax/40IL, Pebax/60IL, Pebax/19.8IL/0.2xGnP, Pebax/18IL/2xGnP, Pebax/16IL/4xGnP, Pebax/32IL/8xGnP, and Pebax/48IL/12xGnP (full names in Table 1).

	Rough face	Smooth face
Pebax	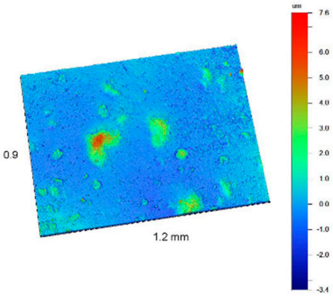	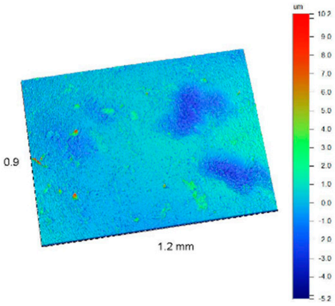
Pebax/20IL	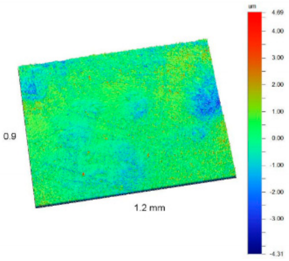	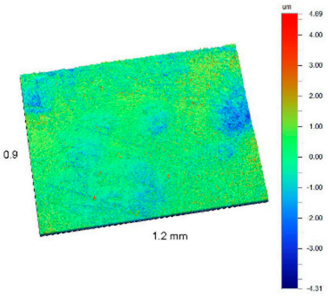
Pebax/40IL	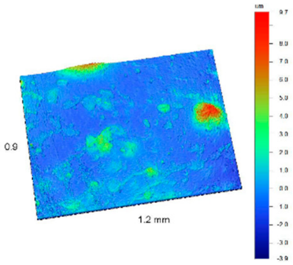	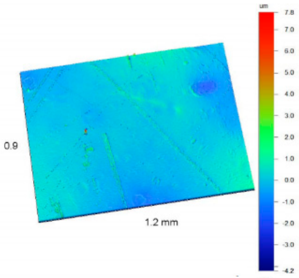
Pebax/60IL	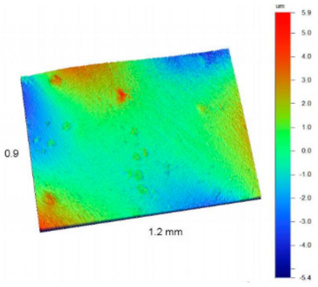	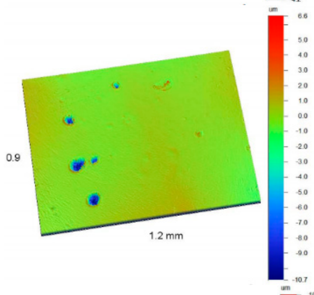
Pebax/19.8IL/0.2xGnP	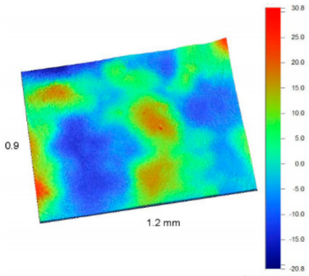	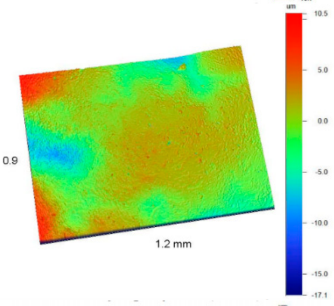
Pebax/18IL/2xGnP	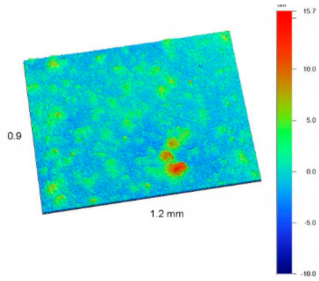	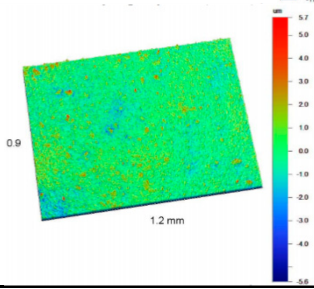

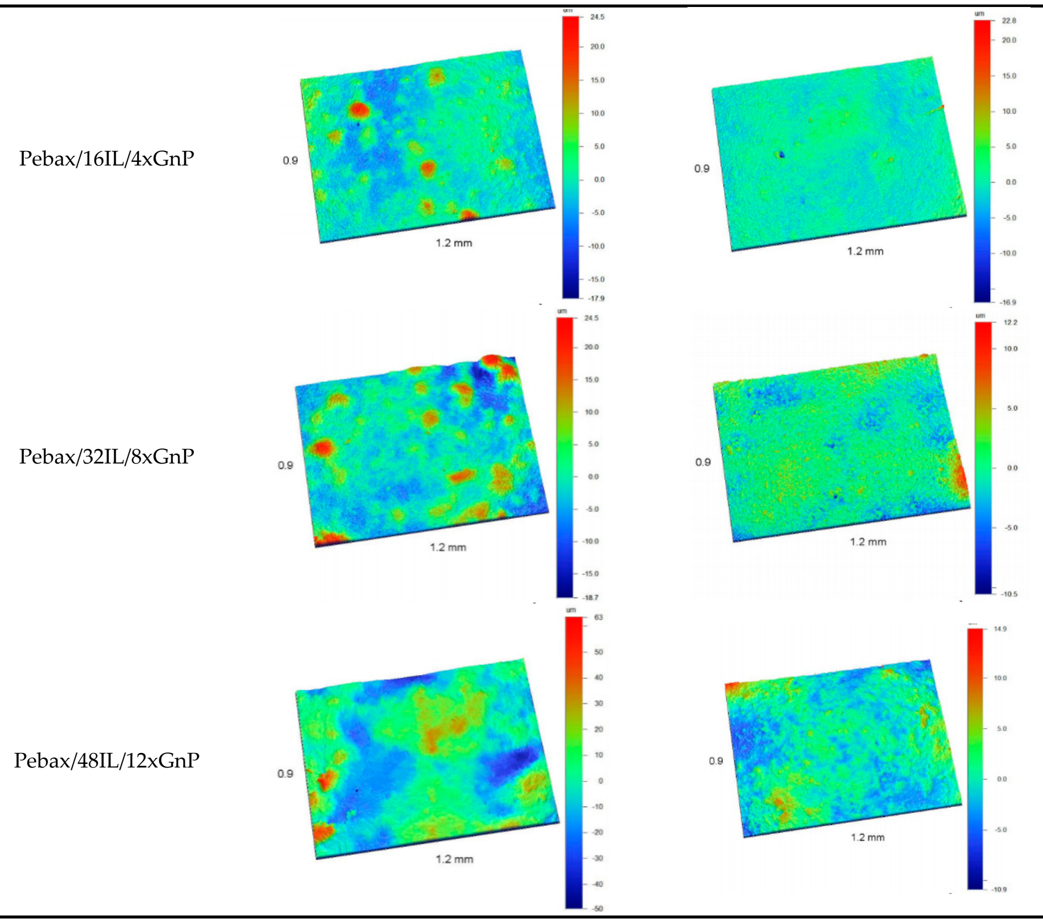

Figure S9. 3D imaging with topographic enhancement of Rough face and Smooth face of Pebax, Pebax/20IL, Pebax/40IL, Pebax/60IL, Pebax/19.8IL/0.2xGnP, Pebax/18IL/2xGnP, Pebax/16IL/4xGnP, Pebax/32IL/8xGnP, and Pebax/48IL/12xGnP (full names in Table 1).

References

S1. Yang, H.; Li, F.; Shan, C.; Han, D.; Zhang, Q.; Niu, L.; Ivaska, A. Covalent functionalization of chemically converted graphene sheets via silane and its reinforcement. *J. Mater Chem.* **2009**, *19*, 4632–4638.

S2. Guo, H.-L.; Wang, X.-F.; Qian, Q.-Y.; Wang, F.-B.; Xia, X.-H. A Green Approach to the Synthesis of Graphene Nanosheets. *ACS Nano* **2009**, *3*, 2653–2659.

S3. Marcano, D.C.; Kosynkin, D.V.; Berlin, J.M.; Sinitskii, A.; Sun, Z.; Slesarev, A.; Alemany, L.B.; Lu, W.; Tour, J.M. Improved Synthesis of Graphene Oxide. *ACS Nano* **2010**, *4*, 4806–4814.

S4. Zhang, L.; Lin, Y.; Xu, S.; Li, R.; Zheng, X.; Zhang, F. Intercalation of perfluorobutane sulfonate into layered double hydroxides. *Applied Clay Science.* **2010,** 48, 641–645

S5. Zhang, K.; Huang, J.; Yu, G.; Zhang, Q.; Deng, S.; Wang, B. Destruction of Perfluorooctane Sulfonate (PFOS) and Perfluorooctanoic Acid (PFOA) by Ball Milling. *Environ. Sci. Technol.* **2013**, 47, 6471−6477

S6. Thanakkasaranee, S.; Kim, D.; Seo, J. Preparation and Characterization of Poly(ether-block-amide)/Polyethylene Glycol Composite Films with TemperatureDependent Permeation. *Polymers*. **2018**, 10, 225
